# High Serum Homocysteine among Women with Polycystic Ovarian Syndrome Visiting an Infertility Clinic of a Tertiary Care Centre

**DOI:** 10.31729/jnma.8411

**Published:** 2024-02-29

**Authors:** Vijay Kumar Sharma, Pratibha Kandel, Sujata Baidya, Smrity Rajkarnikar, Apeksha Niraula, Eans Tara Tuladhar, Aseem Bhattarai, Mithileshwer Raut, Raju Kumar Dubey, Poonam Koirala

**Affiliations:** 1 Department of Clinical Biochemistry, Institute of Medicine, Tribhuvan University Teaching Hospital, Maharajgunj, Kathmandu, Nepal; 2 Department of Obstetrics and Gynaecology, Tribhuvan University Teaching Hospital, Maharajgunj, Kathmandu, Nepal

**Keywords:** *body mass index*, *homocysteine*, *polycystic ovary syndrome*, *prevalence*

## Abstract

**Introduction:**

Polycystic ovary syndrome is a common hormonal disorder that affects women of reproductive age which is characterized by hyperandrogenism, polycystic ovarian morphology, ovarian dysfunction, and hyperinsulinemia. Increased prevalence of cardiovascular disease and higher cardiovascular morbidity is seen in women with polycystic ovary syndrome. This study aimed to estimate the prevalence of high serum homocysteine levels among women with polycystic ovarian syndrome visiting an infertility clinic of a tertiary care centre.

**Methods:**

This was a descriptive cross-sectional study conducted among women with polycystic ovarian syndrome visiting an infertility clinic at the Department of Obstetrics and Gynaecology of a tertiary care centre from 1 June 2023 to 1 September 2023. The study was conducted after obtaining ethical approval from the Institutional Review Committee. Biochemical analysis of gonadal hormones, serum homocysteine and lipid profile was done. A convenience sampling method was used. The point estimate was calculated at a 95% confidence interval.

**Results:**

Among 76 women, the prevalence of high serum homocysteine level was found in 54 (71.05%) (60.86-81.25, 95% Confidence Interval). The mean age of patients was 27.46±6.18 years.

**Conclusions:**

The prevalence of high homocysteine levels among women with polycystic ovarian syndrome is higher than other studies done in similar settings.

## INTRODUCTION

Polycystic ovary syndrome (PCOS) is characterized by hyperandrogenism, polycystic ovarian morphology, ovarian dysfunction, and hyperinsulinemia and is estimated to affect 5-10% of the population.^1^ Increased prevalence of cardiovascular disease and morbidity is seen in women with PCOS.^2^ The Framingham Offspring Study has shown that hyperhomocysteinemia is linked to hyperinsulinemia and may contribute to high blood pressure.^3^ By interfering with the endometrial blood flow and its vascular integrity, homocysteine (Hyc) is hypothesized to disrupt implantation and may increase the risk of an early miscarriage.^4,5^

Homocysteine levels among PCOS are less explored in our setting. So, homocysteine estimation in PCOS will help in risk stratification for cardiovascular disease for unwanted cardiovascular events in the future by instituting lifestyle interventions, diet, and medications in our context.

This study aimed to estimate the prevalence of high homocysteine levels among women with PCOD visiting an infertility clinic of a tertiary care centre.

## METHODS

This was a descriptive cross-sectional study conducted among women visiting the infertility clinic from 1 June 2023 to 1 September 2023 at the Department of Obstetrics and Gynaecology, Tribhuvan University Teaching Hospital (TUTH), Maharajgunj, Kathmandu, Nepal. The ethical approval was obtained from the Institutional Review Committee of the Institute of Medicine, Maharajgunj, Kathmandu, Nepal (Reference number: 540(6-11)E^2^ 079/080). Written informed consent was taken from all the participants. Before being released for analysis, the clinical data were anonymized and de-identified to ensure confidentiality. Patients presenting with PCOS-like features were diagnosed by a gynaecologist via clinical examination and pelvic ultrasonography (USG) based on Rotterdam criteria was included.^6^ Patients with thyroid disease, hyperprolactinemia, adrenal hyperplasia, and cardiovascular disease were excluded. The convenience sampling technique was adopted to enroll the participants. The sample size was calculated by using the following formula:


$$\begin{array}{ll} \text{n} & ={\text{Z}}^{2}\times \frac{\text{p}\times \text{q}}{{\text{e}}^{\text{2}}}\\ & =1.96^{2}\times \frac{0.269\times 0.731}{0.1^{2}}\\ & =76 \end{array}$$

n = minimum required sample size
Z = 1.96 at 95% Confidence Interval (CI)
p = prevalence taken from previous study, 26.92%^4^
q = 1-p
e = margin of error, 10%

The minimum sample size calculated was 76.

The clinico-demographic data were collected at the sample site using a self-designed structured pro-forma and the blood samples were collected on the 2nd day of the menstrual cycle. All the samples were processed in the clinical biochemistry laboratory of TUTH as per standard guidelines. Biochemical analysis of serum luteinizing hormone (LH), follicle-stimulating hormone (FSH), and testosterone was estimated by the chemiluminescence immunoassay technique in the Abbott ci4000 autoanalyzer. Lipid profile: total cholesterol (TC), HDL-cholesterol (HDL-C), LDL-cholesterol (LDL-C), and triglyceride(TG) were estimated by spectrophotometry technique in the Abbott ci4000 autoanalyzer. Homocysteine in the serum sample was quantified using a Biotime BIOT-YF I FIA analyzer based on fluorescence lateral flow immunoassay. The high homocysteine level was defined as elevated homocysteine above 15μmol/L.^7^ BMI was classified according to WHO.^8^

After entering the data in Microsoft Excel 2015, statistical analysis was performed using IBM SPSS Statistics version 22.0. The point estimate was calculated at a 95% confidence interval (CI).

## RESULTS

Among 76 PCOS women, the prevalence of high serum homocysteine level was found in 54 (71.05%) (61.05-81.05, 95% CI). The mean age was 27.46±6.18 years with a minimum of 18 years and a maximum of 40 years. Most of the PCOS women 24 (44.44%) were overweight with a BMI above 25 kg/m^2^ (Table 1). The median BMI was 26.20 (24.50, 30.20) mg/kg^2^.

**Table 1:** BMI categorization among women with high homocysteine levels (n = 54).

Characteristics	n (%)
Underweight	-
Normal	14 (25.93)
Overweight	24 (44.44)
Obese	16 (29.63)

The mean TC was 4.48±0.80 mmol/L (Table 2).

**Table 2:** Anthropometric and biochemical parameters in PCOS population with high homocysteine (n = 54)

Variables	Mean±SD	Median (Q1, Q3)
TC (mmol/L)	4.48±0.80	
HDL-C (mmol/L)		0.90 (0.80, 1.00)
LDL-C (mmol/L)		2.20 (1.80, 3.20)
TG (mmol/L)		1.70 (1.28, 1.90)
LH (IU/ml)		5.48 (4.06, 7.93)
FSH (IU/ml)	4.05±1.60	
LH/FSH ratio	1.70±0.88	
Testosterone (ng/ml)	44.61±14.21	
Homocysteine (μmol/L)	28.35±9.27	

## DISCUSSION

In this study, high homocysteine was found in 71.05%. The finding of the current study was in contrast to the result reported by another study in which, serum homocysteine was high in 36% of the PCOS population.^9^ Similarly, a study conducted in Iran showed that 35.94% of PCOS women had high homocysteine levels.^10^ The finding coincides with the study in which serum homocysteine level was high in 41.1% of PCOS patients.^11^ On the other hand, it was slightly low (26.92%) in the study reported in the Chinese population.^7^ However, the threshold for high and normal levels slightly differed in our study i.e. 15 μmol/L compared to another study i.e. 11 μmol/L.^9^ Because of the atherogenic and prothrombotic characteristics of homocysteine, it is well-established to play a part in cardiovascular morbidity and death. The production of inflammatory cytokines is elevated, oxidative stress is produced, apoptosis is activated, and methylation is defective all of which are molecular pathways of homocysteine-induced cellular dysfunction.^1^

Classification of homocysteine based on BMI in the whole population of our study showed that a higher concentration of homocysteine was present in the obese group as compared to the non-obese group. The increase was more pronounced with increase in BMI. Several possible mechanisms can explain the relationship between homocysteine concentrations and obesity. First, increased Hcy levels caused altered lipids and lipid buildup in tissues, which led to obesity. Second, the deregulation of the cholesterol and triglyceride biosynthesis pathways brought on by Hcy-induced endoplasmic reticulum stress results in abnormal lipid metabolism. Third, obesity is seen as a condition with persistent inflammation. In this regard, increased levels of inflammatory markers (such as CRP and fibrinogen) were discovered to be connected to Hcy levels. Finally, visceral adipose tissue impairs several hepatic activities via the porta by altering the normal functioning of those enzymes responsible for clearing Hcy.^12^

Limitations of the study include a small sample size which may not represent the whole population. Dietary factors and nutritional deficiencies may also alter the level of homocysteine level which was not considered due to the nature of the study design. Further studies are necessary to confirm whether raised Hcy levels in PCOS are a real independent cardiovascular risk factor.

## CONCLUSIONS

The prevalence of high homocysteine levels among women with polycystic ovarian syndrome is higher than other studies done in similar settings. It is recommended to conduct larger-scale studies with diverse populations to confirm the association between elevated homocysteine levels and polycystic ovarian syndrome (PCOS), considering factors such as BMI and dietary influences, to better understand the potential cardiovascular risks associated with PCOS.
